# Prolonged usage of fosaprepitant for prevention of delayed chemotherapy-induced nausea and vomiting(CINV) in patients receiving highly emetogenic chemotherapy

**DOI:** 10.1186/s12885-023-11070-3

**Published:** 2023-07-01

**Authors:** Ai Gao, Shasha Guan, Yinjuan Sun, Lingling Wang, Fanlu Meng, Xia Liu, Liyan Gu, Guo Li, Diansheng Zhong, Linlin Zhang

**Affiliations:** Department of Medical Oncology, Tianjin Medical University General Hospital, No.154, Anshan Dao, Heping District, Tianjin, 300052 China

**Keywords:** Prolonged, Fosaprepitant, Delayed CINV, HEC

## Abstract

**Background:**

Even though chemotherapy-induced nausea and vomiting (CINV) can be well controlled in the acute phase, the incidence of delayed CINV remains high. In this study, we intend to investigate whether prolonged use of NK-1 receptor antagonist (RA) in addition to 5-HT3 RA and dexamethasone (DEX) was more effective in preventing delayed CINV.

**Methods:**

This randomised, open-label, controlled study was designed to compare the efficacy and safety of fosaprepitant 150 mg given on days 1,3 (prolonged group) versus on day 1 (regular group) in patients receiving highly emetogenic chemotherapy (HEC). All patients also treated with palonosetron on day 1 and DEX on days 1–3. The primary endpoint was the incidence of delayed nausea and vomiting. The second endpoint was AEs. All the above endpoints were defined according to CTCAE 5.0.

**Results:**

Seventy-seven patients were randomly assigned to prolonged group and seventy-nine to regular group. Prolonged group demonstrated superiority in controlling delayed CINV to regular group, with statistically significant lower incidence of nausea (6.17% vs 12.66%, *P* = 0.0056), and slightly lower incidence of grade 1 vomiting (1.62% vs 3.80%, *P* = 0.0953) in the delayed phase. In addition, prolonged use of fosaprepitant was safe. No significant difference was found between the two groups regarding constipation, diarrhea, hiccough, fatigue, palpitation and headache in delayed phase.

**Conclusions:**

Prolonged use of fosaprepitant can effectively and safely prevent delayed CINV in patients receiving HEC.

**Supplementary Information:**

The online version contains supplementary material available at 10.1186/s12885-023-11070-3.

## Introduction

Chemotherapy-induced nausea and vomiting (CINV) is the most feared side effect reported for patients receiving chemotherapy [1]. If poorly controlled, CINV can have a deleterious effect on health-related quality of life [2] and compromise treatment adherence [3]. CINV can be categorized into acute phase (0–24 h) and delayed phase (> 24–120 h) [4]. Acute CINV is primarily mediated through serotonin's action on the 5-hydroxytryptamine-3 (5-HT3) receptors in the intestine, while delayed CINV results mainly from substance P acting on the neurokinin-1 (NK-1) receptors in the area postrema and (nucleus tractus solitarius) NTS [5]. For most patients receiving highly emetogenic chemotherapy (HEC), triplet therapy including a NK-1 RA, a 5-HT3 RA and DEX is considered as the basic therapy [6].

Although in most patients, acute CINV can be reasonably controlled with 5-HT3 receptor antagonists, delayed CINV remains a therapeutic challenge. The incidence of delayed CINV is often underestimated. Despite the use of antiemetic prevention, more than 50% of patients undergoing chemotherapy will experience this condition [2]. A prospective, multi-center, multi-country study on cancer patients treated with HEC or MEC showed that 80% of patients who had experienced acute nausea but had no delayed nausea reported that vomiting would not affect their daily life; In contrast, only 56% of patients who experienced delayed nausea but did not have acute nausea reported no effect or very little [7]. Therefore, even if there is no acute CINV, delayed CINV can have a significant negative impact on the daily life of patients.

With the increasing knowledge of substance P in vomiting, NK-1 receptor antagonists have been developed to treat delayed CINV. At present, NK-1 RAs can be obtained through oral and intravenous routes. It is convenient to take aprepitant orally, but failure to adhere to treatment may have a negative impact on the curative effect. Some cancer patients can not tolerate oral therapy, some patients may have difficulty swallowing, and the bioavailability of oral drugs may be reduced due to diarrhea or gastrointestinal ulcers [8]. Fosaprepitant dimethylamine, a phosphorylated analog of aprepitant, is rapidly converted to aprepitant after intravenous (IV) administration [9]. Intravenous injection of fosaprepitant may bring inconvenience to patients and hospital staff, but it ensures the compliance of treatment and is suitable for patients with dysphagia. A phase 3 non-inferiority trial reported no significant difference in CR rate for delayed CINV in patients receiving HEC and antiemesis treatment with ondansetron and dexamethasone in the single-dose fosaprepitant and aprepitant arms [10]. Another phase 3 study evaluating the addition of fosaprepitant to ondansetron and dexamethasone in patients receiving non-AC MEC showed that fosaprepitant significantly improved the incidence of delayed CR (79 vs. 69%; *P* < 0.001) [11]. Based on the trial by Radhakrishnan et al. [12], recommendations have been updated to add fosaprepitant for children who receive HEC or MEC [13]. Therefore, fosaprepitant can be used as a prophylaxis for delayed CINV.

A phase III study for patients receiving 5-day cisplatin-based chemotherapy observed that it is safe and effective to prolong the duration of aprepitant in patients receiving 5-day cisplatin chemotherapy [14]. Considering that the metabolic cycle of fosaprepitant is 48 h, we hypothesized whether the application of fosaprepitant on days 1 and 3 could achieve the control effect of delayed CINV. This randomised, open-label, controlled study compared the efficacy and safety of multi-day and single-day fosaprepitant with palonosetron and dexamethasone as antiemetic prophylaxis in patients receiving HEC.

## Method

### Ethics approval and consent to participate

The experimental protocol was established, according to the ethical guidelines of the Helsinki Declaration and was approved by the Human Ethics Committee of Tianjin Medical University General Hospital. Written informed consent was obtained from individual or guardian participants. They were mentioned in the Result part.

### Study Design and patient selection

Patients were included if their age were 18 years or older with a diagnosis of solid tumor receiving HEC regimen, which includes platinum or anthracycline-based chemotherapy. Patients received carboplatin AUC 5 mg/ml/min i.v. on days 1, cisplatin 75 mg/m2 i.v. on days 1 or epirubicin 90 mg/m2 every 21 days. Patients' Eastern Oncology Collaboration (ECOG) physical status score 0–2 (including boundary values). The regimens were classified as HEC based on NCCN, ESMO and CSCO guidelines [5]. Patients were excluded from the study for mental disorder or if they were taking any prohibited drug (including medicinal marijuana or currently drinking heavily). Patients were also excluded if they were about to receive abdominal or pelvic radiotherapy concurrently with chemotherapy, if they vomited 24 h before chemotherapy, if they had symptomatic brain metastasis, or were receiving systemic corticosteroids chronically.

Patients were randomly assigned to either fosaprepitant 150 mg on day 1 (regular group) or 150 mg once per day on days 1 and 3 (prolonged group). Palonosetron 0.25 mg was administered once per day on days 1. Dexamethasone 6 mg on day 1 and 4 mg once per day on days 2 and 3 was used as baseline antiemetic prophylaxis. Detailed study designs are summarized in Supplementary Fig. 1.

The primary endpoint were incidence of delayed nausea and delayed vomiting. The second endpoint was related AEs. All the endpoints above were defined according to CTCAE 5.0.

### Procedure and data collection

To survey the occurrence of nausea and vomiting, we prepared a nausea/vomiting diary based on the MAT (questionnaire about nausea and vomiting), which was developed by the Multinational Association of Supportive Care in Cancer (MASCC) (Supplementary Fig. 2). The patient diary include the recording of any nausea, vomiting, and adverse effects associated with antiemetic drugs, from day 1 to 14 after chemotherapy. Safety was assessed by collection of adverse events (AEs), including constipation, diarrhea, hiccough, fatigue, palpitation and headache. The use of rescue therapy, defined as any medication taken to treat established nausea or emesis, was also recorded. Patients rated nausea and vomiting daily by using CTCAE 5.0. scale rating their nausea and vomiting for the prior 24 h from no nausea and vomiting to the worst nausea and vomiting with a measurement of score 0 to 4. Nausea symptoms were surveyed and recorded by investigators according to the numeric rating scale (NRS; 4-point scale, in which 1 represents a condition without nausea and 4 represents a condition with the worst conceivable nausea). The results were then categorised into ‘grade 0’ (NRS, 1), ‘grade 1’ (2), ‘grade 2’ (3) and ‘grade 3’ (4). For vomiting symptoms, the number of times vomiting (including dry vomiting) occurred was surveyed every day and evaluated according to the National Cancer Institute-Common Terminology Criteria for Adverse Events Version 4.0 (NCI-CTCAE ver. 4.0). CTCAE grading is the same as nausea. The incidence of nausea and vomiting were assessed during the acute (0–24 h), delayed (24–120 h) and overall (0–120 h) phases after chemotherapy initiation.

### Statistical analysis

According to previous reports and the results of our pre-experiments, we calculated that a sample of 118 patients (59 in regular group; 59 in prolonged group) would provide the study with 80% power to detect a difference between the group proportions of 20% at a two-sided alpha of 0.05. Given an anticipated dropout rate of 20%, total sample size required is 148 (74 in regular group 1; 74 in prolonged group).

Statistical analysis was performed using SPSS software (version 22; SPSS, Inc). Measurement data were expressed as mean ± standard deviation (SD) and were analyzed with t test. Response rates were compared using the chi-square or Fisher’s exact tests. All statistical tests were two-sided, with significance levels at *p* < 0.05.

## Results

### Sample characteristics

A total of 156 patients were enrolled in the study. All the patients were randomly divided into prolonged group (77 patients) and regular group (79 patients). All patients were chemotherapy naive and were evaluated in the first treatment cycle. Table 1 lists the demographic and clinical characteristics of included patients. These characteristics were similar between the two treatment groups. In the regular group, the median age was 64 years old, 39.2% of patients were female, and 62.0% of patients had lung/respiratory cancer. In prolonged group, the median age was 65, 36.4% of patients were women. The most prevalent cancer was also lung/respiratory cancer (70.1%). All patients in each group received HEC.

**Table 1:** Baseline and disease characteristics of patients

	Prolonged group (*n* = 77)	Regular group (*n* = 79)
Gender, %		
Male	63.6	60.8
Female	36.4	39.2
Median age, years	64	65
Cancer type		
Breast	1	3
Lung/respiratory	54	49
Ovarian	1	5
Colorectal	2	0
Gastric	2	1
Esophageal	5	5
Pancreatic	2	1
Other	10	15
Chemotherapy		
Platinum	74	75
Anthracycline	3	4

### Efficacy

The incidence of nausea and vomiting in the acute phase, delayed phase and overall phase were separately compared between two treatment group.

**Fig. 1:**
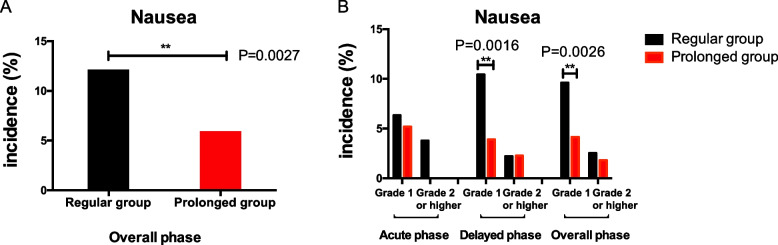
Percentage of patients experiencing nausea

In the overall phase, the proportions of patients experiencing nausea in prolonged group was 5.97%, while that in the regular group was 12.15% (*P* = 0.0027) (Fig. 1A). A similar numerical advantage of prolonged group was also shown in the delayed phases (Fig. 1B). During the delayed phase, the incidence of grade 1 nausea was 3.90%, as compared with 10.44% in regular group (*P* = 0.0016). Prolonged group reduces 6.55% of grade 1 delayed nausea compared to regular group (95%CI: 12.40%-0.68%, *P* = 0.038). 4.16% of patients in the prolonged group experienced grade 1 nausea from day 1 to 14, compared to 9.62% in the regular group (*P* = 0.0026). Therefore, in both the delayed and overall phases, the prolonged group outperformed the regular group in terms of nausea prevention. However, there was no statistically significant difference between groups in the proportions of patients with nausea in the acute phase.

**Fig. 2:**
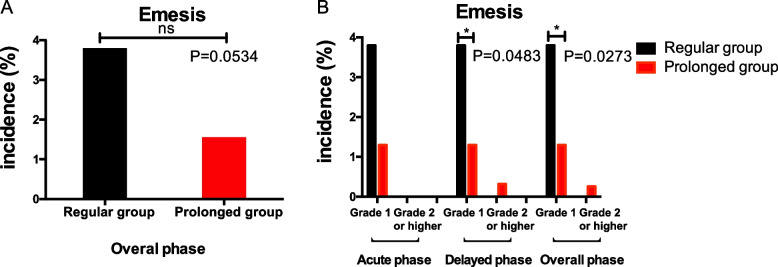
Percentage of patients experiencing vomiting

Multiple-day fosaprepitant regimen was also superior to the regular antiemetic regimen for controlling vomiting in the delayed phase (Fig. 2B), although there was no statistical difference in the overall phase (Fig. 2A). The incidence of vomiting in regular and prolonged group were 3.8% and 1.56% for the overall phase (*P* = 0.0534), although difference between the two groups were not of statistical significance, the incidence of vomiting in prolonged group tended to be slightly lower. The incidence of grade 1 emesis was lower in the prolonged group than in the regular group during the delayed phase (1.30% versus 3.80%, *P* = 0.0483) and overall phase (1.30% versus 3.80%, *P* = 0.0273). Prolonged group reduces 2.52% of grade 1 delayed emesis compared to regular group (95%CI: 2.44%-2.60%, *P* < 0.001). However, the incidence of grade 2 or higher vomiting and the incidence of acute and delayed vomiting were not statistically different between the two groups.

In addition, the incidence of nausea and vomiting beyond delayed phase (120–336 h) was recorded. However, no significant difference was found between two groups (Supplemental Fig. 3).

**Fig. 3:**
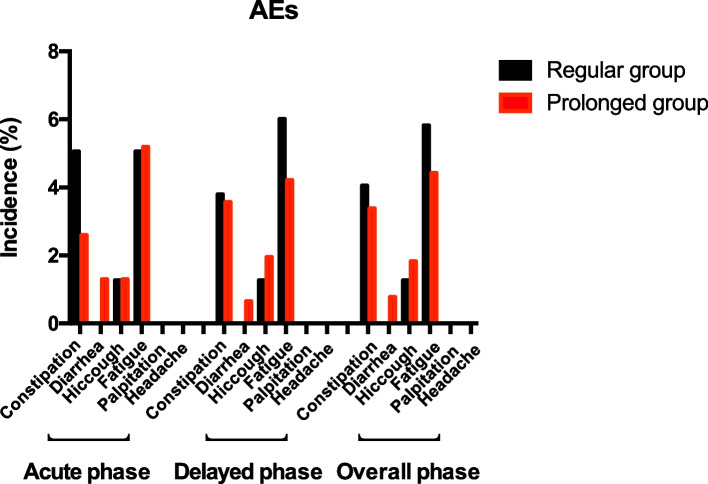
Percentage of patients experiencing AEs

### Safety and tolerability

The most commonly reported AEs of fosaprepitant were constipation, diarrhea, hiccough, fatigue, palpitation and headache [11] (Fig. 3). The overall incidence of AEs was comparable in both treatment groups across the study. Most AEs reported were mild or moderate, with fatigue (prolong group 4.42%, regular group 5.82%, *P* = 0.3730) and constipation (prolong group 3.38%, regular group 4.05%, *P* = 0.6189) being the most commonly reported AEs in both groups. All other AEs had an incidence rate of less than 2%. In comparison to the regular group, there was no evidence of increased AEs and no grade 2 nor more severe AEs were observed in the prolonged group.

### Risk factors

Risk factors for nausea and vomiting symptoms were investigated. The risk factors related with CINV included age, anxiety, nausea with pregnancy, etc. However, these factors showed no statistical significance in the univariate analysis (Table 2).

**Table 2:** Risk factors for nausea and vomiting symptoms

	Univariate analysis	
	Odds ratio (95% CI)	*P* value
Age(< 60 years old)	1.487 (0.558–3.962)	0.428
Anxiety	0.497 (0.077–3.224)	0.464
The patient slept less than 7 h on the night before chemotherapy	2.181 (0.920–5.169)	0.077
Nausea with pregnancy	1.540 (0.455–5.207)	0.487
Antiemetic therapy was performed outside the hospital after the last cycle of chemotherapy	1.621 (0.816–3.220)	0.168
CINV occurred after last cycle of chemotherapy	0.992 (0.698–1.410)	0.964
The patient received the second cycle chemotherapy	1.176 (0.929–1.488)	0.178
The patient received ≥ 3 cycles of chemotherapy this time	1.158 (0.975–1.376)	0.094

## Discussion

To the best of our knowledge, this is the first study to provide efficacy and safety data on a multiple-day course of fosaprepitant combined with a 5-HT_3_ RA and a corticosteroid for the prevention of CINV in patients receiving HEC. For the primary endpoint, we compared the incidence of delayed nausea and vomiting in patients treated with HEC between the single and multiple-day fosaprepitant administration groups, and reported significant inter-group differences of grade 1 nausea (10.44% vs 3.90%, *P* = 0.0016) and grade 1 emesis (3.80% vs 1.30%, *P* = 0.0483) in the delayed phase, suggesting a benefit of multiple-day fosaprepitant administration during the delayed period post-chemotherapy. The low incidence of grade 1 nausea was notable given the unmet clinical need for CINV prevention in the delayed setting, particularly for nausea control. Our current study reported high rates of “no nausea” in regular group and prolonged group (87.34% vs 93.83%, *P* = 0.0041, data not show) during delayed phase, indicating a potential quality-of-life (QoL) benefit. Overall, our new antiemetic regimen outperformed the standard control antiemetic regimen in the delayed phases of CINV associated with HEC.

For acute nausea and vomiting symptoms seen after chemotherapy, no difference in incidence was found between the two treatment groups. Consistent with previous research results, the acute phase is mainly mediated by 5-HT_3_ receptors and is therefore particularly sensitive to 5-HT_3_ receptor antagonists [15].In our study, acute CINV can be effectively controlled by the use of palonosetron, which has a high affinity for binding to 5-HT3 receptors. Panolosetron also inhibits cross-talk between the NK-1 and 5-HT3 receptor pathways [16], which is assumed to be the cause of the observed effect in preventing delayed nausea and vomiting. As a result, our findings revealed that panolosetron and fosaprepitant act synergistically to keep the overall incidence of nausea (≤ 12%) and vomiting (≤ 4%) at a low level. So our research demonstrated the advantages of maintaining superior CINV management in overall phases with this antiemetic combination.

Our findings demonstrate the efficacy of multiple-day fosaprepitant in preventing CINV, particularly in the delayed phase, which has a significant negative impact on a patient’s daily life [5]. A prospective, multi-center, multi-national study compared the impact of acute and delayed CINV on patients’ QoL after MEC or HEC [7] and discovered that CINV continues to adversely affect the QoL of patients who did not experience acute nausea. Our new antiemetic combination reduces the occurrence of delayed CINV, thereby improving patients’ QoL after chemotherapy and their willingness to undergo the next chemotherapy cycle, which is beneficial chemotherapy completion overall survival.

It is worth noting that antiemetic trials typically evaluated CINV control for 120 h (in the acute phase and delayed phase). An observation study, on the other hand, reported the presence of a certain number of patients who developed CINV after 120 h, implying the importance of monitoring for beyond delayed CINV that develops after 120 h [17]. In our study, the efficacy assessment was extender until 336 h (14 days), and we founded that single administration of fosaprepitant was non-inferior to 2-day administration of fosaprepitant in controlling CINV beyond 120 h.

In our study, the regimen of adding an extra day of fosaprepitant was generally well tolerated, and no new safety signals were found when compared to previous fosaprepitant studies [10, 18]. AE profiles for the two treatment regimens were similar and typical for a cancer population undergoing chemotherapy [19]. Most AEs in both groups were mild, less than 5%. Furthermore, neither treatment raised any concerns about cardiac safety.

It is worth noting that fosaprepitant has been reported to be associated with a high frequency of injection site reactions (ISRs), leading to clinical problems [18]. Fosnetupitant is an injectable phosphorylated prodrug of netupitant. A recent phase III study demonstrated non-inferiority of fosnetupitant to fosaprepitant and demonstrated fosnetupitant has the potential to overcome the risk of developing ISRs with fosaprepitant administration [20]. Thus, fosnetupitant will be valuable in the prophylaxis of delayed and beyond delayed CINV. In addition, the application of peripherally inserted central catral catheters (PICC) and totally implantable venous-access ports (TIVAP) in chemotherapy significantly reduces the risk of ISRs.

Our new antiemetic combination targeting two critical antiemetic pathways, was safe, well tolerated and highly effective of HEC. It should be noted that the categories of emetics is only based on the incidence of acute CINV, not delayed or overall CINV [5]. A recent study found that the chemotherapy regimen are inconsistent predictor of delayed CINV [21]. Our research is limited to patients treated with HEC, so we need to conduct prospective large sample randomized clinical trials on patients treated with MEC and HEC in the future to confirm this.

In conclusion, among patients receiving HEC regimen, prolonged use of fosaprepitant is effective and safe in preventing delayed CINV.

## Supplementary Information

**Additional file 1: Supplementary figure 1.** CONSORT diagram.

**Additional file 2:Supplementary figure 2.** Patient dairy.

**Additional file 3:Supplementary figure 2.** Percentage of patients experiencing nausea and vomiting.

## Data Availability

The datasets generated and analysed during the current study are not publicly available, but are available from the corresponding author on reasonable request.

## References

[1] Griffin AM (1996). On the receiving end. V: Patient perceptions of the side effects of cancer chemotherapy in 1993. Ann Oncol.

[2] Cohen L (2007). Chemotherapy-induced nausea and vomiting: incidence and impact on patient quality of life at community oncology settings. Support Care Cancer.

[3] Hesketh PJ (2008). Chemotherapy-induced nausea and vomiting. N Engl J Med.

[4] Navari RM, Aapro M (2016). Antiemetic Prophylaxis for Chemotherapy-Induced Nausea and Vomiting. N Engl J Med.

[5] Rapoport BL (2017). Delayed Chemotherapy-Induced Nausea and Vomiting: Pathogenesis, Incidence, and Current Management. Front Pharmacol.

[6] Aogi K (2021). Optimizing antiemetic treatment for chemotherapy-induced nausea and vomiting in Japan: Update summary of the 2015 Japan Society of Clinical Oncology Clinical Practice Guidelines for Antiemesis. Int J Clin Oncol.

[7] Bloechl-Daum B (2006). Delayed nausea and vomiting continue to reduce patients' quality of life after highly and moderately emetogenic chemotherapy despite antiemetic treatment. J Clin Oncol.

[8] Kraut L, Fauser AA (2001). Anti-emetics for cancer chemotherapy-induced emesis: Potential of alternative delivery systems. Drugs.

[9] Lasseter KC (2007). Tolerability of fosaprepitant and bioequivalency to aprepitant in healthy subjects. J Clin Pharmacol.

[10] Grunberg S (2011). Single-dose fosaprepitant for the prevention of chemotherapy-induced nausea and vomiting associated with cisplatin therapy: randomized, double-blind study protocol–EASE. J Clin Oncol.

[11] Weinstein C (2016). Single-dose fosaprepitant for the prevention of chemotherapy-induced nausea and vomiting associated with moderately emetogenic chemotherapy: results of a randomized, double-blind phase III trial. Ann Oncol.

[12] Radhakrishnan V (2019). Intravenous fosaprepitant for the prevention of chemotherapy-induced vomiting in children: A double-blind, placebo-controlled, phase III randomized trial. Pediatr Blood Cancer.

[13] Hesketh PJ (2020). Antiemetics: ASCO Guideline Update. J Clin Oncol.

[14] Albany C (2012). Randomized, double-blind, placebo-controlled, phase III cross-over study evaluating the oral neurokinin-1 antagonist aprepitant in combination with a 5HT3 receptor antagonist and dexamethasone in patients with germ cell tumors receiving 5-day cisplatin combination chemotherapy regimens: a hoosier oncology group study. J Clin Oncol.

[15] Aapro M (2005). 5-HT(3)-receptor antagonists in the management of nausea and vomiting in cancer and cancer treatment. Oncology.

[16] Shirley M (2021). Netupitant/Palonosetron: A Review in Chemotherapy-Induced Nausea and Vomiting. Drugs.

[17] Tamura K (2015). Testing the effectiveness of antiemetic guidelines: results of a prospective registry by the CINV Study Group of Japan. Int J Clin Oncol.

[18] Saito H (2013). Efficacy and safety of single-dose fosaprepitant in the prevention of chemotherapy-induced nausea and vomiting in patients receiving high-dose cisplatin: a multicentre, randomised, double-blind, placebo-controlled phase 3 trial. Ann Oncol.

[19] Gralla RJ (2014). A phase III study evaluating the safety and efficacy of NEPA, a fixed-dose combination of netupitant and palonosetron, for prevention of chemotherapy-induced nausea and vomiting over repeated cycles of chemotherapy. Ann Oncol.

[20] Hata A (2022). Randomized, Double-Blind, Phase III Study of Fosnetupitant Versus Fosaprepitant for Prevention of Highly Emetogenic Chemotherapy-Induced Nausea and Vomiting: CONSOLE. J Clin Oncol.

[21] Jordan K (2014). International antiemetic guidelines on chemotherapy induced nausea and vomiting (CINV): content and implementation in daily routine practice. Eur J Pharmacol.

