# Supplementary figures and images for: Prolonged usage of fosaprepitant for prevention of delayed chemotherapy-induced nausea and vomiting(CINV) in patients receiving highly emetogenic chemotherapy
Source: BMC Cancer. 2023 Jul 1;23:609. doi: 10.1186/s12885-023-11070-3 (PMC10314477; doi:10.1186/s12885-023-11070-3)

Supplementary figure 1. CONSORT diagram.

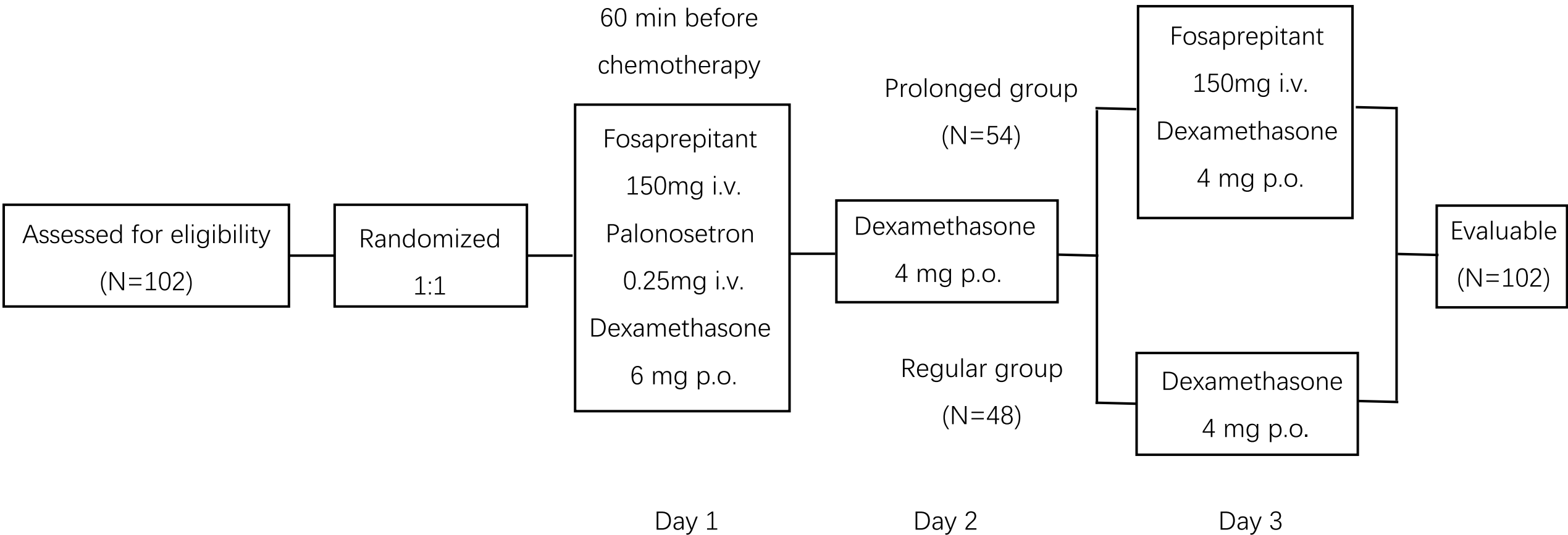

Supplement: Supplementary file 1 — Additional file 1: Supplementary figure 1. CONSORT diagram. [file 12885_2023_11070_MOESM1_ESM.pdf]

Supplementary figure 2. Percentage of patients experiencing nausea and vomiting.

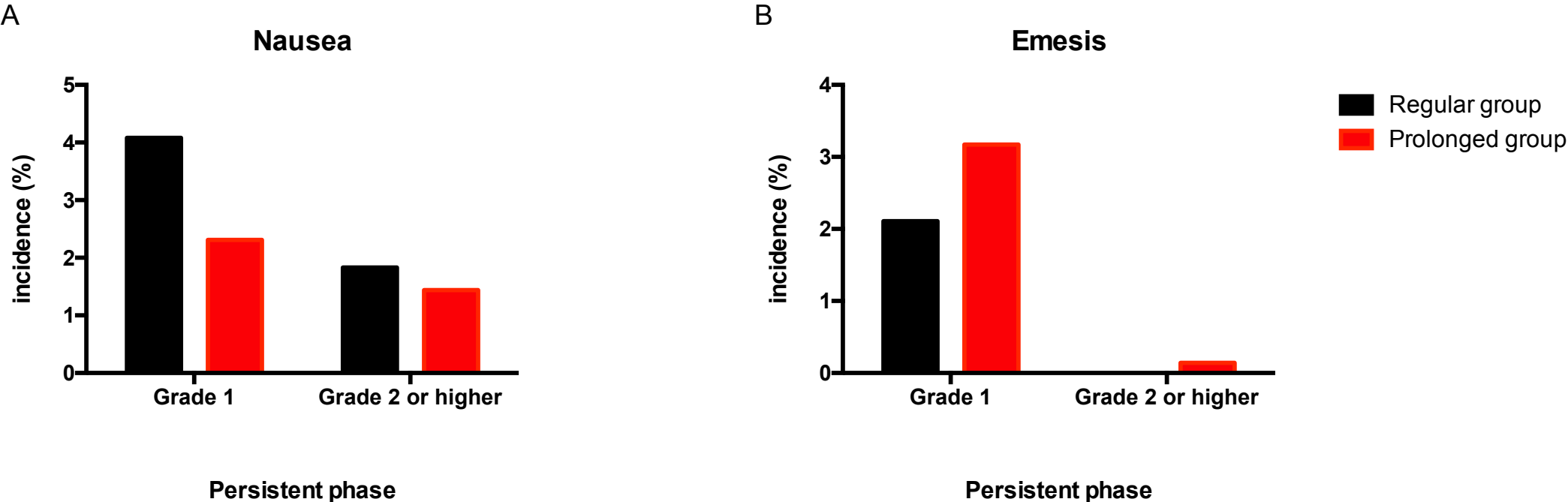

Supplement: Supplementary file 3 — Additional file 3:Supplementary figure 2. Percentage of patients experiencing nausea and vomiting. [file 12885_2023_11070_MOESM3_ESM.pdf]
