# Supplementary material for: Prolonged usage of fosaprepitant for prevention of delayed chemotherapy-induced nausea and vomiting(CINV) in patients receiving highly emetogenic chemotherapy
Source: BMC Cancer. 2023 Jul 1;23:609. doi: 10.1186/s12885-023-11070-3 (PMC10314477; doi:10.1186/s12885-023-11070-3)
Supplement: Supplementary file 2 — Additional file 2:Supplementary figure 2. Patient dairy. [file 12885_2023_11070_MOESM2_ESM.pdf]

Supplementary figure 2. Patient dairy

	D1	D2	D3	D4	D5	D6	D7	D8	D9	D10	D11	D12	D13	D14
Nausea														
Vomiting														

Please fill in the table with the appropriate number in reference to the explanation.

- Nausea

Please choose the poorest degree of nausea over the course of 1 day.

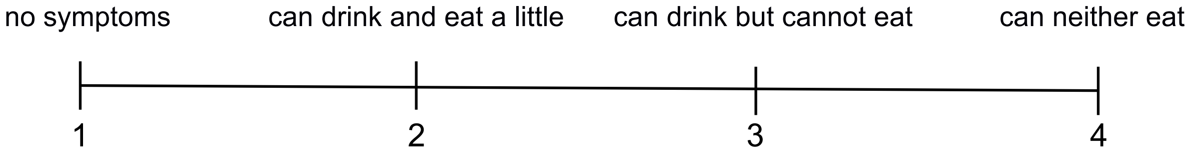

- Vomiting

Please count the number of times that vomiting continued for more than 5 minutes over the course of 1 day.

1. No vomiting occurred
2. 1-2 times
3. 3-5 times
4. More than 6 times
